# Phenotyping photosynthesis: yes we can

**DOI:** 10.1093/jxb/erad496

**Published:** 2024-02-02

**Authors:** Samuel H Taylor

**Affiliations:** Lancaster Environment Centre, Lancaster University, Lancaster LA1 4YQ, UK

**Keywords:** Chlorophyll fluorescence, genome-wide association study, GWAS, LIFT, MultispeQ. phenotyping, PhotosynQ, photosynthesis

## Abstract

This article comments on:

Keller B, Soto J, Steier A, Portilla-Benavides AE, Raatz B, Studer B, Walter A, Muller O, Urban MO. 2024. Linking photosynthesis and yield reveals a strategy to improve light use efficiency in a climbing bean breeding population. Journal of Experimental Botany 75, 901–916.

**For crop improvement, identifying natural genetic variants that impact photosynthesis and demonstrating their utility for breeding programmes has been a missing link (** **Theeuwen *et al.,* 2022** **). Keller *et al.* (2024) show how higher throughput tools can break down these barriers, with the promise that photosynthetic phenotypes and their underpinning genetics can increasingly be made accessible for crop breeding programmes.**

Photosynthesis marks the interface between photon energy and biochemical energy conservation in living systems, and solar rhythms of day and night are deeply enmeshed with the workings of crop productivity (Steed *et al.*, 2021). Outside of a lab environment, solar progression sets the pace for photosynthesis, and when we survey genetically diverse crop populations in the field we recognize time as a constant enemy, with photosynthesis tracking the ebb and flow of photosynthetic photon flux (Long *et al.*, 2022). We rise before dawn to measure reference states (Maxwell and Johnson, 2000), and we suffer in the sun to take advantage of the midday period when crop photosynthesis consistently hits its high notes (Long *et al.*, 1996). We fret about how recently leaves had a good dose of sun (Logan *et al.*, 2007). Is the leaf facing the sun? Have we unnecessarily shaded it while making our previous measurement? Sometimes, to get control, photosynthetic tissues, with or without stems/roots/soil, are extracted from the field before dawn and the day is spent coddling and calming them with carefully dosed pre-treatments, encouraging peak activity at a time of our choosing (Ferguson *et al.*, 2023). By using controlled cuvettes, we can stabilize light input, but the longer a leaf stays in a cuvette, the more we are paranoid about physiological responses to the spectral composition of light (McClain and Sharkey, 2020), temperature, and boundary layer properties affecting energy balance, and changing concentrations of carbon dioxide and water (Busch, 2018). The fluid complexity of photosynthetic responses to the environment means that even high-throughput, rapid ‘survey’ mode phenotyping measurements often do not attempt to completely determine ‘true’ leaf operational states; instead, fixed cuvette settings are commonly used to capture standardized snapshots of photosynthesis that can facilitate meaningful comparisons among genotypes.

Control freakery in photosynthetic physiology arises because photosynthesis is a suite of highly responsive physicochemical processes, built to cope with solar input that flickers between famine and feast, not only at daily time scales but also momentarily because of self-shading and variable sky conditions (Kaiser *et al.*, 2018; Slattery *et al.*, 2018; Long *et al.*, 2022). Evidence suggests that steady-state photosynthesis (captured by traditional light and CO_2_ response curves, or midday survey measurements in sun-exposed leaves) shows patterns of heritability distinct from non-steady-state photosynthesis (Cruz *et al.*, 2016; Acevedo-Siaca *et al.*, 2021). Is it therefore possible to phenotype photosynthesis under genuinely variable, natural conditions and obtain results useful to plant breeders?

## Controlled conditions and the quantitative genetics of photosynthesis

Critical contributions have been made to dissection of the genetics of photosynthesis using controlled-environment (CE) systems automated to obtain pulse amplitude modulation (PAM) chlorophyll fluorescence phenotypes at high frequency (Rooijen *et al.*, 2015; Cruz *et al.*, 2016; Oakley *et al.*, 2018; Prinzenberg *et al.*, 2020). Measurements of photosynthetic efficiency (quantum yield of PSII, Φ_PSII_) and quenching of chlorophyll fluorescence by photochemical and non-photochemical processes are relatively straightforward using PAM fluorescence, and systems can be designed to snapshot momentary responses to dynamic irradiance across large collections of genotypes (Cruz *et al.*, 2016). The Arabidopsis model is critical for these experiments because of the space constraints imposed by CE systems and its support for advanced genetics. The excellent work in this area targets a future where gene information from Arabidopsis can be rapidly translated to genomic predictions or identification of orthologous major effect loci in crops, and those opportunities will come. For resource-limited crop improvement programmes today, especially in the global south or where breeders are working with orphan crops, a climate crisis that is hastening obsolescence of current varieties (Challinor *et al.*, 2016) means that crop breeders interested in photosynthesis need easier wins.

## For now we see through a glass darkly: but then shall we see face to face

While leaf-level measurements using PAM chlorophyll fluorescence are routine, it is exceptionally difficult to repeatably generate the necessary saturating flashes in a high-throughput field situation. Consequently, in parallel with PAM measurements in CE systems, researchers have been advancing remote sensing methods that promise to bring genuinely high-throughput, high-frequency measurements to crop systems. Solar-induced fluorescence (SIF) is a rapidly developing and promising technology, with devices available for hand-held, tower-mounted, or airborne imaging at scales relevant to agronomic trials and breeder plots, but it is so far better understood at the ecosystem scale (Siebers *et al.*, 2021). By contrast, the laser/light-induced fluorescence transient (LIFT) method exploited by Keller *et al.* (2024) is a more mature technology from the perspective of crop field trials. It can be tuned to provide measurements at the level of individual plants/plots, and paired with spectral reflectance measurements that can add information about features including chlorophyll content (Soares *et al.*, 2021; Zendonadi dos Santos *et al.*, 2021; Keller *et al.*, 2022).

By far the most affordable tools for phenotyping photosynthesis in the field are hand-held, open source, MultispeQ PAM fluorometers (Kuhlgert *et al.*, 2016). Like earlier systems used in research labs over many decades, MultispeQ measures individual leaves. Keller *et al.* (2024) show that MultispeQ measurements made in field plots of common bean at Darién, Colombia, support a significant association between the Φ_PSII_ response to the photosynthetic photon flux rate (PPFR) (Response_G:PPFR_; Box 1) and a marker on chromosome 9. That marker was pleiotropic for yield in larger field trials (including trials in Tanzania and Uganda) carried out within the same collaboration, and was associated with elevated statistical significance in MultispeQ measurements from separate trials in Palmira, Colombia, and in glasshouse LIFT experiments in Germany. Critically, Keller *et al.* (2024) demonstrated that phenomic prediction, based on early season measurements of Response_G:PPFR_, outperforms genomic prediction of yield/biomass in environments where multi-annual yield/biomass data are lacking. This suggests a route to using measurements of photosynthesis to drive efficiencies in multi-environment breeding programmes, selecting the best lines early and reducing the number of lines carried through to harvest.

Box 1. **What is Response_G:PPFR_?**

**Fig. 1.**
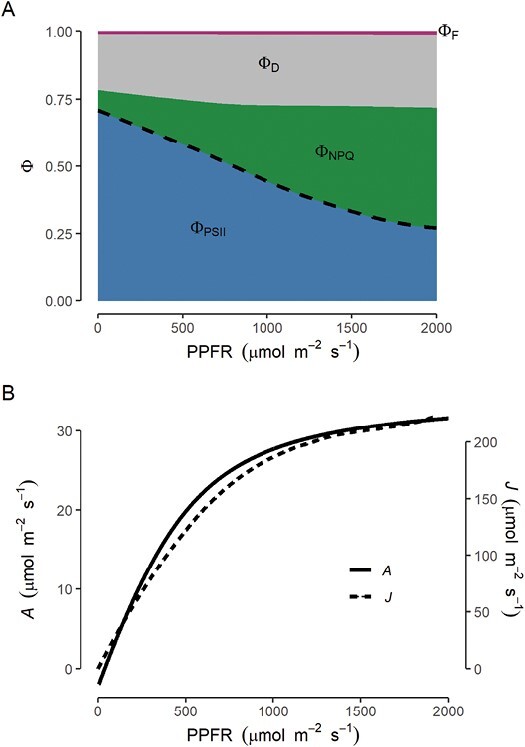
Response of (A) quantum yield of PSII (Φ_PSII_) and (B) net CO_2_ assimilation rate (*A*) to photosynthetic photon flux rate (PPFR). Quantum yields for competing processes in chloroplast light harvesting are: Φ_PSII_, PSII photochemistry; Φ_NPQ_, non-photochemical quenching; Φ_D_, constitutive thermal dissipation; and Φ_F_, fluorescence (Hendrickson *et al.*, 2004, assuming that Φ_F_ ~0.01). Trends are based on a high to low PPFR, rapid light response curve measurement drawn from a published dataset (Taylor *et al.*, 2022), and *J* is approximated assuming fixed values of α=0.84, and β=0.5.


The phenotype exploited by Keller *et al.* (2024) captures photosynthetic response to light (photosynthetic photon fluence rate, PPFR) throughout a period of crop growth and development. The measured aspect of photosynthesis is the quantum yield of PSII (Φ_PSII_). Φ_PSII_ corresponds to the proportion of photosynthetically active quanta of light reaching chlorophyll molecules associated with PSII: the first reaction centre in the chloroplast linear electron transport chain, where quanta are used to drive the famous water splitting reaction evolving O_2_, H^+^, and the electrons used to drive carbon reduction in CO_2_ assimilation. In a PAM fluorometer, Φ_PSII_ is determined for a photosynthesizing leaf as the variable chlorophyll fluorescence stimulated by a several-fold greater than ambient, saturating flash of light, a technical feature that limits its application in field and glasshouse settings. By contrast, LIFT stimulates variable fluorescence as a result of cumulative pressure from a series of exceptionally fast sub-saturating pulses, making it possible to apply the method in field settings (Keller *et al.*, 2019). Using either method, Φ_PSII_ approaches a maximum of ~0.83 after dark adaptation (Maxwell and Johnson, 2000), with the remaining ~17% of absorbed energy being dissipated through a small contribution from fluorescence and a larger loss due to inherent inefficiencies in energy transfer and slow repair of photosystem damage incurred in the course of normal operation. In Fig. 1A, these alternative energy dissipation pathways are assigned quantum yields following Hendrickson *et al.*, (2004): Φ_D_ corresponding to constitutive dissipation; Φ_F_, fluorescence. The combination of Φ_D_ and Φ_F_ shows limited variation with PPFR. Decreases in Φ_PSII_ observed in light-adapted leaves and as PPFR increases are primarily explained by non-photochemical quenching (Φ_NPQ_ in Fig. 1A), a series of mechanisms that act to minimize additional damage to reaction centres by increasing the fraction of absorbed energy that is dissipated as heat (Maxwell and Johnson, 2000;

Logan *et al.*, 2007). Response_G:PPFR_ determined by Keller *et al.* (2024) represents the genotype-specific difference in slope of the Φ_PSII_–PPFR relationship. Increasing PPFR offsets decreasing Φ_PSII_, so the rate of electron transport for photosynthesis (*J*=α·PPFR*·*βΦ_PSII_, where α is absorption and β the ratio of electrons from PSII reaching acceptors such as NADPH per photochemical event) increases with PPFR, roughly corresponding to the rate of net CO_2_ assimilation (*A*; Fig. 1B). Comparing across different plants and longitudinally through the development of those plants, as in the study of Keller *et al.* (2024), Response_G:PPFR_ is a compound trait potentially influenced by time-varying responses of leaf absorptance (depending on, for example, pigment composition/amount and leaf angle), distinct forms of non-photochemical quenching, alternative electron sinks, and potentially feedback from limitations imposed on photosynthesis by diffusive properties of leaves such as stomatal and mesophyll conductance (Murchie and Lawson, 2013). In the context of complex genetic control over photosynthesis, phenomic prediction using Response_G:PPFR_ is likely to benefit from this phenotype being an integrated outcome of a broad suite of mechanisms. Correspondingly, the genetic associations of Response_G:PPFR_ may be distinct from those influencing singular aspects of photosynthetic performance.

## The offer

Problem solved—Keller *et al.* (2024) have shown us how measurements of photosynthetic phenotypes in the field can contribute to drive yield improvement in a key crop for global food security. Yes but… photosynthesis researchers will still be control freaks. We still need to carefully define measurement conditions. We still need to sneak up on plants with the sun in our faces to avoid unnecessary shading of leaves. We still need to respect that day-by-day and hour-by-hour shifts in sunlight, temperature, humidity, and soil and plant water status will shift our target. Following the example of Keller *et al.* (2024), we also need to take thoughtful account of those factors in genetic analyses. Our offer to crop breeders interested in photosynthesis is not yet behold, I give unto you power, but we can reasonably say: yes, we can help you with that, and there’s more to come.
